# Identity, individuality and indistinguishability in physics and mathematics

**DOI:** 10.1098/rsta.2022.0109

**Published:** 2023-09-18

**Authors:** Gabriel Catren

**Affiliations:** Laboratoire SPHERE (UMR 7219), Université Paris Cité - CNRS, 8 Rue Albert Einstein, Bâtiment Olympe de Gouge, 75013 Paris, France

**Keywords:** quantum physics, gauge theories, homotopy type theory, identity, individuality, indistinguishability

## Abstract

In this brief survey, we discuss some of the scientific and philosophical problems and debates that underlie the notions of *identity*, *individuality* and *indistinguishability* in physics and mathematics. We critically analyse the different positions for or against the existence of indistinguishable objects in different scientific theories, notably quantum mechanics and gauge theories in physics and homotopy type theory in mathematics. We argue that the different forms of indistinguishability that occur in many areas of physics and mathematics—far from being a problem to be eradicated—exhibit a rich formal structure that plays a key role in the corresponding theories that needs to be properly understood.

This article is part of the theme issue ‘Identity, individuality and indistinguishability in physics and mathematics’.

## 1. Introduction

The notions of *identity*, *individuality* and *indistinguishability* play crucial roles in philosophy, physics and mathematics [1,2].^1^ Whereas *identity* can be understood as a logical notion associated with the reflexive character of the relation of equality defined by the equal sign =, *individuality* is a more philosophical notion that makes reference to the fact that an individual entity is, in Lowe’s terms, ‘the single object that it is [⋯] distinct from others’ [3, p. 75]. On the other hand, the notion of (*in*)*distinguishability* makes reference to the fact that an entity might be endowed with certain properties by means of which it can (or not) be distinguished from other entities (for a discussion of these different notions see [4]).

The notions of *identity*, *individuality* and *indistinguishability* as well as their interrelations elicit in particular the following questions:

— Is identity a primitive relation (as Frege maintained^2^) or can it be defined in terms of other predicates, as in the Hilbert–Bernays definition? With regard to this question and related matters, see [6], [7, §5], [8, pp. 63–64], [9, pp. 12–15] and [10].
— Is identity a universal notion or is it always relative to a given ontological domain [11–15]?
— Is it possible and/or necessary to conceive an ontology (or a philosophical logic) without a predicate of identity [1]? Is it possible to philosophically conceptualize and mathematically formalize the notion of *non-individual*? What is the relationship between indiscernibility and non-individuality? Does indiscernibility imply non-individuality or can we conceive indiscernible individuals?
— Should individuality be defined
  — exclusively in terms of properties (as claim the defenders of the so-called *bundle theory*),
  — in terms of the spatio-temporal localization of the corresponding individuals (which can be included in the previous case if spatio-temporal localization is understood as a property),
  — or by appealing to some form of haecceity, Lockean substance, ‘primitive thisness’ [16] or ‘transcendental individuality’ [17]?
— Can we accept the existence of differences *solo numero*, i.e. of numerical differences not grounded on qualitative differences [18]? In McTaggart’s terms, is the (numerically) diverse necessarily (qualitatively) dissimilar [19, ch. X, pp. 95–101]?
— How should we understand the mathematical notion of equality ‘=’? Should it be understood in intentional or in extensional terms (e.g. the equality of functions)? As definitions and/or as propositions with a truth value? As expressing numerical equality or indiscernibility? As a relation of synonymy between names or as a relation between the denoted entities?

In physics, the ability to individualize, distinguish and reidentify particles and other physical systems through time, and across different contexts, seems to be crucial for understanding their behaviour (see [1] and references therein). However, there is still much to be explored and understood about how, if, and under which conditions these notions can be defined in certain central chapters of contemporary physics, notably in *quantum mechanics* and the *gauge theories* of fundamental interactions. In the case of quantum mechanics, the statistics of identical particles challenge the understanding of the relations between identity, individuality and indistinguishability inherited from classical physics (see §3 below). In particular, are quantum systems self-identical and distinguishable individuals? If not, what are the ontological categories that are needed to describe quantum systems? In the case of gauge theories such as general relativity and Yang–Mills theories, the fundamental role played by (local) symmetries seems to challenge the idea according to which indiscernible configurations should be simply—as Leibniz famously argued in the correspondence with Clarke [20]—identified (see §4 below). In particular, are gauge symmetries a mechanism to control the representational redundancy associated with the existence of different coordinate systems or do they encode some deep fact about the ‘logic of nature’ [21]? Are gauge symmetries just the consequence of a mathematical ‘surplus structure’ (Redhead) [22], a mere ‘descriptive fluff’ (Earman) [23]? Can we simply get rid of these ‘ghosts’ (Wigner) [24] or do they play a fundamental role that has yet to be properly understood?

In mathematics, equality propositions of the form a=b are fundamental and omnipresent components of mathematical discourse. Even if we might expect that the significance of such a basic building block of mathematics should be clear by now, the understanding of equalities is still under discussion. This problem acquired more relevance since the development of *category theory* (see for instance [25]) and, more recently, of *homotopy type theory* [26,27]. Very briefly, these theories convey different forms of generalizations of the relation of equality beyond the strict set-theoretic equality, like for instance the notions of *isomorphism* and *equivalence of categories* in category theory or the type-theoretic notion of *propositional equality* in homotopy type theory (see §5 below). These reconceptualizations of the mathematical notion of equality have been considered a ‘revolution’ in the foundations of mathematics that questions set-theoretic foundations. How does this radical shift reshape our understanding of the notions of identity, individuality and distinguishability? Is Leibniz’s principle of the identity of indiscernibles (PII) still valid in this new setting? What consequences does this revolution have on the aforementioned debates in the foundations of physics?

The aim of this collection is to survey several aspects of the state of the art and the open debates regarding our understanding of the notions of *identity*, *individuality* and *indistinguishability*, in both contemporary physics and mathematics. In this introductory article, we present a general overview of the questions that guide the research in these topics, and the different strategies to deal with them—making a special mention of the works included in this volume. We start by reviewing Leibniz’s PII in §2. Next, we jump into the problem of quantum indistinguishability in §3, where we also discuss the non-standard logical frameworks that were inspired by this peculiar properties of quantum systems. In §4, we deal with the problem of gauge transformations and the problem of surplus mathematical structure in physical theories. In §5, we provide a brief overview of the debates about identity and indistinguishability in cutting edge areas of current mathematics. Finally, in §6, we draw some conclusions.

## 2. Leibniz’s principle of the identity of indiscernibles

An underlying philosophical theme that connects the foundations of physics and mathematics is the discussion of the validity of Leibniz’s PII (see [18] and references therein). According to this principle two entities that are qualitatively identical (that is, indistinguishable) are also numerically identical, that is, one and the same entity. In other terms, there are no two things that share all their properties. In this way, Leibniz’s PII forbids the existence of differences *solo numero*, i.e. of distinct individuals that are perfectly similar, of *numerical differences* that are not grounded on *qualitative differences*. Formally, Leibniz’s PII can be formulated in second-order logic as follows:

2.1 ∀x∀y[∀P(P(x)⇔P(y))⇒x=y].

Leibniz’s principle is at the core of the philosophical discussions concerning the role played by the notions of identity, individuality and discernibility in physics and mathematics. First, it should be stressed that the validity of the PII depends on the kind of properties included in the range of the universal quantifier ∀P in (2.1). For instance, including properties of the form x=a (where a denotes a given individual) makes the PII trivially true. Moreover, the validity of the PII might also depend on the significance of the equality symbol ‘=’. Indeed, if the equality relation is weakened (or stretched) from strict (or numerical) equality to indiscernibility, then the PII becomes either a mere tautology, or an explicit definition of the equality relation *qua* indiscernibility [28].

If the PII holds, then entities can be completely individualized by specifying all their properties, which is certainly an appealing possibility. This is of special relevance for *bundle theories*, which state that individuals are nothing but bundles of properties, possibly related by a relation of *compresence* [29, Part IV.8]. By defining individuals as bundles of properties, these theories get rid of the metaphysical presupposition of an underlying substratum that would carry or instantiate these properties. Bundle theories find their origins in the work of British empiricists of the eighteenth century such as Berkeley and Hume and was later resumed by Russell [30, pp. 97–98] and Ayer ([31], [32, p. 42]). In other words, the PII provides a *principium individuationis* exclusively based on properties (see Lombardi [33] for a bundle theory approach to the problem of defining individuality in quantum mechanics). Besides its philosophical appeal, Leibniz’s PII can be used to define identity along the Hilbert–Bernays approach [6,7]. One of the main problems is that Leibniz’s PII seems to be deprived of any form of logical or conceptual necessity (unless we include properties of the form x=a [34]). Indeed, a standard avenue to refute the validity of Leibniz’s PII has been the conceptual construction of counter-examples given by possible worlds containing numerically different and indiscernible entities (like Black’s universe containing two similar spheres [35]). Moreover well-established physical theories like quantum mechanics provide empirically attested counter-examples. It is worth noting that Leibniz himself maintained that the PII is a metaphysical principle which is only valid for entities defined by ‘complete notions’. This means that it cannot be extrapolated to abstract or ‘incomplete notions’ (see for instance [36, p. 32]).

On the other hand, accepting that the PII might not hold seems to be a possibility that many scholars would prefer to avoid. An interesting symptom of the resistance to relinquish the PII is provided by the debates in philosophy of mathematics around the objection proposed by Burguess and Keränen to mathematical structuralism [37–42] (see also Wüthrich’s translation of this *identity problem for realist structuralism* to the framework provided by space–time physics [43–45]).^3^ On the physical side, the attempt to preserve Leibniz’s PII at all costs led some scholars to follow Quine [46] in the introduction of different *grades* (absolute, relative and weak) *of discernibility* [47–49] (see also [50,51]). Very briefly, these authors maintain that Leibniz’s PII can be forced to be valid by relaxing or weakening the corresponding notion of discernibility (by including for instance irreflexive relations). In turn, it has been counterargued that these weaker forms of discernibility just restate the fact that the corresponding multiplicity of individuals (e.g. points in a homogeneous space) is purely numerical, without grounding these numerical distinctions on qualitative differences (as the ‘spirit’ of Leibniz’s PII seems to require) [52–54]. From a more philosophical standpoint, the willingness to preserve Leibniz’s PII at all costs results (at least partially) from the fact that if the PII were not valid, then the individuality of an entity would rely on some form of metaphysical notion of *primitive thisness* [16], *haecceity*, *bare* or *thin particular*, *Lockean substratum* or (in Post’s terms) *transcendental individuality* [17]. All these notions make reference to some form of nonqualitative support that would carry the corresponding properties and provide an ante-predicative form of individuation. In Post’s terms, transcendental individuality ‘means something that transcends observable differences’ [15,17]. According to the scholars that understand individuals as bundles of properties, this possibility seems to be a mere metaphysical presupposition that we should better avoid.^4^

## 3. Indistinguishability in quantum physics

Besides other fundamental features of quantum mechanics—such as *superposition* (and thereby *indeterminacy*), *contextuality* and *entanglement*—the study of *quantum indistinguishability* became a central subject of research in the foundations of quantum theory literature [55]. This topic gave rise to debates that range from philosophical stances associated with different interpretations of the quantum formalism [52,56–65], to more technical issues, like the development of entanglement measures for indistinguishable particles [66–73] or the characterization of quantum indistinguishability as a resource in quantum information theory [74,75]. It is worth stressing that the advent of a second wave of quantum technologies allowed physicists to perform robust manipulations of individual quantum systems. Many of the ‘gedanken’ experiments discussed by the founding fathers of quantum theory in the past can now be analysed under the light of very accurate tests. Thus, there is no escape: the ‘shut up and compute’ attitude is no longer an option for many working physicists trying to understand things like indistinguishability, entanglement and contextuality among other puzzling features of quantum theory. A short but self-contained review of the different problems and possible solutions for dealing with the entanglement of indistinguishable quantum systems is presented by Majtey *et al.* [76]. Regarding the harnessing of quantum indistinguishability as a resource see Piccolini *et al*. [77]. For a more philosophical perspective on the problem of entanglement of identical particles see [33,78].

Quantum systems can be classified in two big classes: bosons and fermions. While the former have integer intrinsic spin and can occupy the same quantum state, the latter possess half-integer spin and two of them cannot occupy the same state.^5^ In particular, the Pauli exclusion principle forbids that two electrons have the same set of quantum numbers. This principle can be naturally derived from the symmetrization postulate in standard quantum mechanics [80], but it can also be understood as a consequence of the spin-statistics theorem in quantum field theory [81]. The symmetrization of the states for aggregates of ‘identical’ quantum systems leads to the well-known Fermi–Dirac and Bose–Einstein statistics, which have many empirically well-tested consequences (like for instance superconductivity [82]). To date, there is no known experimental violation of the symmetrization postulate at the fundamental level (see for example [83] for a very accurate experiment). Since these properties of quantum systems determine the way in which atomic levels are filled, they play a key role in explaining the structure of matter.

But there is more. The notions of identity and individuality become problematic when we try to apply them to multiparticle quantum systems of the same kind. The problem is that there are experimental situations in which there is no operational procedure to distinguish between (for instance) two photons or two electrons (see de Barros & Holik [84], where some simple examples are discussed in an introductory manner). Even worse, in certain cases, there is no robust operational way to reidentify a quantum system at different moments of time, as if a quantum system were not subject to the constraint of continuous existence that characterizes macroscopic objects.^6^ Once a photon enters into an aggregate of indistinguishable photons, there is no meaningful way to reidentify it. The fact that these features lead to problems concerning the notions of identity, sameness and individuality was soon recognized by Born [86] and Schrödinger [85],^7^ and later stressed by Post [17] and Manin [88] among others. Now, should we push their analyses further and try to conceive (and even mathematically formalize) a rigorous notion of *non-individual* or the notions of identity and individuality might still be applicable to quantum systems (at least in certain regimes or approximations)? In fact, the landscape of possible interpretative positions about the status of the notions of identity, individuality and indistinguishability in quantum physics is organized by a massive cleavage between the so-called *received view*—based on the thesis that quantum particles of the same type are absolutely indiscernible (which implies for some authors their non-individuality) [1,34]—and the alternative views [78,89].

Whereas Born [86] and Schrödinger [87] clearly stated that quantum particles do not behave as individuals, Post argued that ‘non-individuality has to be introduced right at the start’ (rather than introducing individuality by using labels and then wiping it out by using symmetries under permutations of the labels) [17, p. 19]. Later on, Manin argued that standard set theory might not provide an adequate formal framework to work with aggregates of ‘identical quantum particles’ [88, p. 36].^8^ Then, Krause and collaborators took seriously the challenge of ‘developing a totally new [formal] language’ to deal with quantum non-individual ‘particles’. In Quine’s terms, a non-individual is an entity without identity, that is, an entity a for which the proposition a=a does not apply. These ideas fuelled the development of non-standard set-theoretic frameworks inspired in quantum theory and based on non-reflexive logics (see [1, Ch. 6 & 8]). In this context, the standard notion of identity is not a primitive one, and the axioms are chosen in such a way that the objects at stake mimic collections of indistinguishable quantum systems. The most widely discussed of these approaches is *quasi-set theory* (see [34] and references therein). Very briefly, quasi-set theory formalizes the notion of a collection of indistinguishable entities that lack self-identity. In particular, the non-standard features of quasi-set theory were used to develop an alternative approach to the description of quantum systems by constructing a Fock space that no longer uses the symmetrization of states to mimic quantum statistics [90]. While quasi-set theory is focused on quantum non-individuality, other approaches such as *quaset theory* capture other features of quantum mechanics, such as indefinite properties [91]. A quantum mereology—in which the notion of quantum uncertainty and undefined number of components are related to logical undecidability—was also proposed in [92] (see also [93]).

It is worth addressing here the relation between the notions of *non-individual* and *indistinguishability* in the conceptual framework provided by Leibniz’s PII. Indeed (as mentioned above), if we accept properties of the form x=a, then Leibniz’s PII is trivially true, which means that indistinguishable entities are forbidden (briefly, each entity a is unambiguously distinguished by the property of being identical to itself). Hence, we can hold space for indistinguishable entities by questioning Quine’s thesis that there is ‘no entity without identity’ [94, p. 23]. Formally, this can be done by suspending the universal application of the mathematical relation of equality and, *a fortiori*, that of self-identity. Since expressions of the form x=a might no longer be applicable, the trivial validity of Leibniz’s PII is blocked. All in all, an ontology in which self-identity is not necessarily a well-formed proposition can accommodate indistinguishable entities.

Taking sides with the received view, de Barros & Holik [84] claim in their contribution that quantum indistinguishability is not only a crucial and independent feature of quantum systems—besides entanglement and superposition—but also that it is deeply connected with the notions of quantum interference and contextuality. In their contribution they discuss simple (but well-established) experiments under this light, and analyse several of the so-called ‘quantum paradoxes’ from the standpoint provided by an ontology of non-individuals. According to their analysis, most of the problems discussed in the foundations of quantum theory implicitly assume that the entities at stake are individuals, that is, entities obeying the classical laws of identity. They argue that the motivations underlying Bell’s theorem and the assumptions that lead to the Kochen–Specker contradiction do not follow if the assumption that quantum systems are individuals is dropped. By using an ontology based on (quantum) bundles of properties in which the PII is not valid, Lombardi’s contribution analyses the problem of entanglement of indiscernible quantum systems [33] (see also [95]). Lombardi’s main claim is that many of the philosophical problems that appear when analysing entanglement of indiscernible particles are dissolved when we consider them in the light of an ontology of non-individuals. The contribution by Becker Arenhart [4] analyses the interpretation of quantum entities in terms of non-individuals from a different perspective. The author defends the position that quantum entities are better described as *nomological objects*, that is, as classes of objects but not as single individuals. According to this author, this explains why the theory fails to provide tools to decide whether quantum entities are individuals or not. With this move, the author claims, the problem of individuality disappears, since it is out of the scope of the theory.

At the opposite side of the aforementioned cleavage, Bigaj [78] and Dieks [89] propose different types of critique of the received view. On the one hand, Dieks denies that the notion of particle is more than just a convenient approximation valid in certain regimes. From the standpoint of quantum field theory, the notion of a (semi-classical) particle associated with the Fock space representation is an emergent concept which does not belong to the fundamental ontology (which seems more suitably described by using a notion of ‘one individed physical whole’ associated with a quantum field). In the general case of a coherent superposition of (anti)symmetrized product states, the concept of particles is simply not relevant. But when the notion of particle is applicable, particles are distinguishable (defined by one particle state) and possess physically defined identities. In this way, the received view is challenged by assuming that in the last instance quantum systems are not (individual or non-individual) particles. On the other hand, Bigaj analyses in his contribution how the components of a composite system can be individuated by using physical properties represented by projectors rather than by relying on labels (see also [57]). He goes on to argue that the concept of particle might still be applicable in the case of entangled states in a manner that depends on the selection of a particular individuating framework (like a projective decomposition of the identity) or on some form of physical process like decoherence. In the wake of the works by Ghirardi and co-workers [96–98], Bigaj also analyses the relation between the notion of entanglement and the non-factorizability that merely arises from the (anti)symmetrization of a product state. In Kastner [99], a notion of *quantum haecceity* is proposed in the framework of Kastner’s *transactional interpretation* of quantum theory. This author maintains that the exchange of labels associated with a symmetrization of the wave function should be understood as an exchange of a quantum form of ‘transcendental individuality’ or ‘primitive thisness’ that Kastner calls *quantum haecceity*. According to Kastner, quantum haecceity endows quantum systems with a ‘quasi-individuality’ that encodes the potential for different outcomes in a measurement process. In turn, such physical measurement processes entail the distinguishability of the different components.

Another avenue to question the thesis that indistinguishable entities should be understood as non-individuals—and to defend the thesis that the notion of *indistinguishable individuals* makes sense (see for instance [56])—is to exclude predicates of the form x=a from the formulation of Leibniz’s PII. This restriction can be justified by arguing that the property x=a (whose unique truthmaker is a) just encodes the numerical distinction between a and other entities, without grounding this numerical distinction on a qualitative dissimilarity.^9^ Moreover, a is just a label denoting an entity. If the corresponding entity is equipped with discerning properties, then we can just use these properties to individualize a instead of the predicate x=a. Equivalently (along the lines of the descriptivist theory of names), we can *define* the label a as a shorthand for the individualizing bundle of properties of the corresponding entity. If a is indistinguishable from other entities, then the predicate x=a discerns the entity at stake only when we define the reference of the label a by means of a demonstrative act or indexical sign. Hence, the individuality of such an entity still relies on some form of primitive thisness. All in all, the argument according to which the predicate x=a defines a discerning property can be soundly put into question. If we exclude this predicate from the universal quantifier in (2.1), then the PII is no longer trivially valid. We can then hold space for indistinguishable entities without relinquishing the notions of equality and self-identity.

In order to conclude this section, it is also worth noting that *quantum* indistinguishability might be considered as a particular form of indistinguishability which differs from the classical indistinguishability associated with different classical objects that share all their properties, like Black’s indistinguishable spheres [35] or Boltzmann particles. Indeed, we can in principle distinguish two different forms of indistinguishability. What we could call *classical indistinguishability* refers to entities (a) that are perfectly alike and (b) that are endowed with some form of ‘transcendental individuality’ or ‘primitive thisness’ [17] (see also [1, Sect. 1.3]).^10^ As Post argues, this form of classical indistinguishability underlies Boltzmann statistics, in which configurations related by an exchange of indistinguishable parties are counted as different [17, p. 15]. By contrast, (what we could call) *quantum indistinguishability* might be understood as a stronger (or ‘absolute’) form of indistinguishability in which entities (a) are perfectly alike and (b) lack ‘transcendental individuality’ or ‘primitive thisness’. This could explain why configurations related by an exchange of indistinguishable particles are counted as one configuration. The situation is somehow similar to what happens in gauge theories, in which configurations related by a gauge symmetry define one and the same *physical* (or coordinate-independent) configuration in the *reduced phase space* of the theory (see §4). The analogy could be pushed further by noting that in both cases the corresponding symmetries (the exchange symmetries and the gauge symmetries, respectively) play a similar role, namely that of depriving the corresponding labels or coordinates of any intrinsic physical meaning. In this way, both exchange and gauge symmetries remove representational redundancy, ‘surplus structure’ (Redhead [22]) or ‘descriptive fluff’ (Earman [23]) (see [103] and [79, pp. 25–35] for such a gauge-oriented interpretation of exchange symmetries).^11^ Rather than introducing non-physical labels together with a formal mechanism intended to deprive the labels of any physical meaning, we could prefer an intrinsic formalism that avoids using such ‘surplus structure’ right from the start (like the Fock space formalism in quantum field theory or the reduced phase space description in gauge theories). However—as we shall see in the next section—the thesis that fundamental physical symmetries like exchange symmetries or gauge symmetries are nothing but a mere formal device by means of which we can get rid of a previously introduced representational redundancy is not devoid of problems.

## 4. Indistinguishability in gauge theories

The notion of symmetry is intimately related to the notion of indistinguishability. Briefly, a symmetry transformation is a non-trivial operation acting on a given configuration such that the initial and the final states are indistinguishable. It is a well-known fact that symmetries play an essential role in physics [104,105]. A fundamental step in the understanding of the physical importance of the notion of symmetry was the discovery by Emmy Noether of the relation between (global) *symmetries* (defined by a finite dimensional Lie group) of the corresponding Lagrangian and *conserved quantities* (i.e. quantities that do not change under the temporal evolution of the system). Noether’s second theorem encodes a generalization of this result to local symmetries, that is, to symmetries defined by an infinite-dimensional Lie group [106–108]. In this case, the existence of local symmetries leads to the existence of relations between the canonical variables of the Hamiltonian formulation called *constraints* [109–111]. The importance of Noether’s results cannot be overestated since it provides the mathematical foundations of one of the most important achievements of the twentieth century physics, namely the geometrization of the fundamental interactions. Whereas general relativity provides a geometric description of the gravitational interaction, Yang and Mills successfully extended—in the wake of previous work done by Weyl—this geometrization programme to the other fundamental interactions, namely, electromagnetism and the string and weak nuclear forces [112,113]. The mathematical structures of these theories share a common formal feature, namely, the presence of so-called *gauge symmetries* given by the invariance of the physical predictions under *local gauge transformations*, that is, under space–time-dependent transformations of the local coordinate systems (for the formulation of general relativity as a gauge theory see [114] and references therein). The geometrization of the fundamental interactions programme was then crowned by the recognition that these interactions can be modelled by means of a beautiful mathematical theory, namely the theory of (Ehresmann and Cartan) connections on principal fibre bundles [114].

The problem of understanding the epistemic and/or ontological scope of the notion of *gauge symmetry* is one of the main conceptual issues posited by these theories (see for instance [21–23,53,115–122]). We could summarize the conundrum posited by gauge symmetries by saying that the presence of symmetry transformations which by definition do not produce any observable effect seem to have nonetheless direct (or at least indirect) empirical significance (some examples are listed below). Also here the landscape of philosophical interpretations of gauge symmetries is organized around two massive opposing views, namely the position which has been called the *received view* (based on the thesis that gauge symmetries do not have direct empirical significance) [121], and the corresponding alternative views. On the one hand, the presence of gauge symmetries is understood as a mere consequence of the mathematical ‘surplus structure’ [22] resulting from the fact that a unique physical configuration can be described by using different coordinate systems [116,119,120].^12^ In Witten’s terms, ‘gauge symmetries are redundancies in the mathematical description of a physical system rather than properties of the system itself’ [124]. According to this argumentative line, the act of choosing a particular gauge adds non-physical structure, and the corresponding gauge symmetry guarantees that the physical or ‘observable’ predictions do not depend on this arbitrary choice (i.e. that they are invariant under gauge transformations). It would always be possible—at least in principle—to project the coordinate-dependent description to a coordinate-independent one in which all the redundant ‘surplus structure’ is removed out (see for instance the notion of *reduced phase space* in [109,111]). According to the received view, the fact that a coordinate-dependent description might be useful to perform certain calculations should not blind us to the fact that the election of such a description is only a matter of convenience.

According to the critics of the received view, the presence of gauge symmetries cannot be reduced to a mere redundancy of the mathematical representation, but rather encodes some deep fact about the ‘logic of nature’ [21] that has to be properly understood [53,118,125]. The main argument to justify the search for a deep meaning of gauge symmetries is that these symmetries seem to have non-trivial physical consequences, notably

(1) the relation between gauge symmetries and fundamental physical interactions encoded in the heuristic *gauge principle* [113] (see also [21,117,121,126,127]),^13^
(2) the role played by gauge symmetries in *renormalization theory* [128],
(3) the role played by gauge symmetries in the definition of the so-called *topological solutions*—like instantons—associated with fibre bundles that are topologically non-trivial,
(4) and the supposed ‘physical reality’ of the gauge potential in the Aharonov–Bohm effect and similar escenarios [129–134].

In order to try to unravel the mysteries of gauge symmetries, different research avenues have been explored. Rovelli, for instance, argues that gauge symmetries are a manifestation of the relational nature of physical observables.^14^ In turn, Greaves and Wallace use Galileo’s ship thought experiment [135] to argue that local symmetries can have empirical significance or observable consequences [118] (see also [136–140]). Very briefly, the empirical significance of a symmetry would result from the fact that a transformation acting on a subsystem of the universe might have non-trivial observable effects associated with the relation between the subsystem and the reference system provided by the corresponding environment.^15^ A different line of argumentation clings to the fact that a symmetry transformation produces by definition states that are, strictly speaking, indiscernible. However, this strict indiscernibility cannot be used as an argument in favour of the thesis that gauge symmetries are mere mathematical redundancies since the very group(oid)-theoretical structure of these indiscernibilities is an essential feature of the corresponding theory. The epistemic fact that a given physical configuration can be described by means of different coordinate systems is a consequence of an intrinsic geometric structure that characterizes the theory at stake. For instance, the fact that observables in relativistic physics in flat space–time must be Poincaré invariant is not a mere epistemic constraint that removes the ‘surplus structure’ given by the existence of different frames of reference. Poincaré invariance rather results from the fact that the underlying space–time is assumed to have a particular symmetry, which is an intrinsic property of this particular space–time [53].^16^ It is also worth noting that the most sophisticated mathematical formalism for dealing with gauge symmetries—namely, the so-called *BRST formalism* [111]—does not proceed by removing the gauge symmetries, but rather by unfolding the higher structure that they convey. Rather than removing the degrees of freedom that are nothing by ‘pure gauge’ (at least according to the received view), the BRST formalism increases the original number of variables by introducing the so-called *ghosts*, *ghosts of ghosts*, and so on and so forth.^17^

All in all, the problem of analysing the significance of physically indistinguishable configurations in gauge theories remains an open and fruitful field of research in both physics and philosophy of physics.

## 5. Revisiting mathematical equality

Let’s consider now the role played by the notions of *identity*, *individuality* and *indistinguishability* in mathematics. Of course, the notion of *equality* (and *a fortiori* the notion of identity) is fundamental in mathematics.^18^ To a first approximation this notion has a seemingly paradoxical character: whereas equality propositions of the form a=b state—in a somewhat contradictory manner—that two different things are equal, identity propositions of the form a=a state—in a somewhat tautological manner—that a thing is identical to itself. This paradoxical nature of the notion of equality has been stressed by Wittgenstein in the following terms: ‘[⋯] to say of two things that they are identical is nonsense, and to say of one thing that it is identical with itself is to say nothing at all’ [143, §5.5303]. But if this is so, then we could ask with Quine ‘[o]f what use is the notion of identity if identifying an object with itself is trivial and identifying it with anything else is false?’ And Quine answers, ‘[⋯] the useful statements of identity are those in which the named objects are the same and the names are different, it is only because of a peculiarity of language that the notion of identity is needed. If our language were so perfect a copy of its subject matter that each thing had but one name, then statements of identity would indeed be useless.’ [144, pp. 208–209]. In other terms, the only non-trivial use of the notion of identity would be according to Quine that of encoding the relation of *synonymy* between linguistic expressions.

Quine’s stance seems to fall short of what is required to understand even elementary mathematical statements like 27×37=999. Since the two different expressions compute to the same value 999, we could say that they are literally *equivalent*. But of course, 27×37 and 999 are different *qua* arithmetic expressions since they convey different computational contents. The use of the symbol = can then be understood as an abuse of notation resulting from the fact that the two expressions yield the same numerical value after performing the corresponding computation. In other terms, 27×37 and 999 are in the same equivalence class defined by the equivalence relation ‘two arithmetic expressions a and b are equivalent if they yield the same numerical value after computation’. In Frege’s terms, we could say that 27×37 and 999 express different *senses* (or *modes of presentation*) of the same reference. Indeed, by introducing the Fregean distinction between *sense* and *denotation*, Girard describes the situation in the following terms: ‘This equality [27×37=999] makes sense in the mainstream of mathematics by saying that the two sides denote the same integer [⋯]. This is the denotational aspect, which is undoubtedly correct, but it misses the essential point: There is a finite *computation* process which shows that the [references] are equal. It is an abuse [⋯] to say that 27×37 *equals* 999, since if the two things we have were *the same* then we would never feel the need to state their equality. Concretely we ask a *question*, 27×37, and get an *answer*, 999. The two expressions have different *senses* and we must *do* something (make a proof or a calculation [⋯]) to show that these two *senses* have the same [*reference*].’ [145, pp. 1–2]. Is it possible to make this kind of abuse of notation rigorous? Is it possible to define a notion of equality that explicitly encodes this computational dimension? Is it possible to introduce a notion of equality that somehow takes into account Fregean distinction between sense and reference?

Now, the notion of mathematical equality (and, *a fortiori*, the notion of identity) has undergone a far-reaching process of reconceptualization that started with the development of *category theory*, continued with the enhancement of the latter to higher category theory, and has recently entered into a new phase with the development of *homotopy type theory* in the early new millennium [26,27] (see also [28] and references therein). Category theory made clear that the notion of strict equality is indeed too strict. This fact motivated the ‘stretching’ of the notion of strict equality into the notion of *isomorphism*. It soon became clear that a full-fledged development of this weakening of the notion of strict equality requires us to extend category theory to higher category theory. In turn, we could say that homotopy type theory focuses on the sector of higher category theory that encodes this extension of the notion of equality, namely the higher categories known as *∞-groupoids*. Very briefly, the main new ingredient of homotopy type theory is the structure given by the so-called *propositional equality* between terms of a type. Given two terms of a type a,b:X, the propositional equality $a{=}_{X}b$ is itself a type whose terms are the concrete identifications between a and b, that is, the proofs that a and b are equal (which means that the proposition is false if the type $a{=}_{X}b$ is empty). Since two proofs $p,q:a{=}_{X}b$ of a propositional equality are not necessarily equal (i.e. the *uniqueness of identity proofs* principle does not hold), a propositional equality $a{=}_{X}b$ might envelop a complex structure of higher identifications. It follows that a type has the structure of an ∞-groupoid which—according to the so-called *homotopy hypothesis*—can be understood as a geometric object known as *homotopy type*.

The main ontological stance at the base of homotopy type theory—namely that the fundamental mathematical objects are given by ∞-groupoids or homotopy types—can be understood as the most refined development to date of the history that starts with Galois’ introduction of the notion of group in the beginning of the nineteenth century (see [146] for a conceptual discussion of Galois theory). By addressing the problem of finding solving formulae for polynomial equations, Galois was confronted with the existence of solutions to polynomial equations of the form p(x)=0 (with p(x)∈K[x], where K is a field) such that no K-relation could discern them. Galois’ breakthrough was the invention of a new mathematical notion—the notion of *group*—which encoded the structure defined by the indiscernible solutions. In this way, group theory was born as a mathematical ‘*theory of ambiguity*’ (as Galois himself dubbed it [147, p. 94]) that formalizes the limits of a given arithmetic language (the field K) to discern solutions of polynomial equations over K. In Galois theory, groups encode the structure of that which cannot be ‘said’ in a given arithmetic language, namely the numerical difference between solutions that are indiscernible with respect to that language.^19^ The lesson that we can draw from this story that begins with Galois theory and culminates (at least for the moment) with homotopy type theory is that differences that make no difference might carry nonetheless a rich mathematical structure. In order to conclude this section, it is also worth noting that the problem of deciding whether Leibniz’s PII holds or not in homotopy type theory is still under discussion (see [28,148] for two different approaches to this question).

## 6. Conclusion: who is afraid of indiscernibles?

In this brief survey, we have analysed different ways in which the notions of identity, individuality and indistinguishability are used in physics and mathematics, and revisited some of the philosophical questions they elicit. In order to conclude this survey, we will take the risk of making abstraction of the details of the different local debates and types of indistinguishabilities that we have described—both in the foundations of quantum and gauge physics and in the foundations of mathematics—and try to focus on (what we think is) an emerging global pattern. Interestingly enough, we can recognize a sort of unique cleavage that traverses these different research areas, which is defined by the attitude towards the existence of indiscernibles.

On one side of this cleavage, there is a tradition that tries to foreclose by all means the existence of indiscernibles. Since indiscernibles are associated with differences that make no difference—e.g. gauge symmetries, exchanges of identical particles, substitutions *salva veritate*, differences *solo numero*—it is tempting to consider them as a symptom of a metaphysical or representational ‘surplus structure’ that should be removed at some point. A possible strategy consists in denying the very existence of indiscernibles by looking for—eventually hidden—distinguishing properties (as is done in some approaches to quantum theory) or by introducing, in the wake of Quine, weaker grades of discernibility. Other strategies (like in certain interpretations of gauge theories) proceed by considering the presence of indiscernibles as a symptom of a representational redundancy. In mathematics, different proofs of the same proposition, different computations that yield the same result or different intensional definitions of the same object (like different mathematical expressions that define the same functional correspondence between the domain and the codomain) also appear to be a mere surplus structure that we can discard at some point. In Wittgenstein’s terms, we could safely ‘throw away the ladder after [we have] climbed up it’ [143, §6.54]. According to this argumentative line, the progress of scientific understanding would move in the direction of recognizing the merely descriptive, representational or even subjective nature of this type of ‘surplus structure’. Symmetry principles, Leibniz’s PII or different forms of extensional truncations would allow us to distinguish the essential, intrinsic or empirically accessible content from what Earman called ‘descriptive [or constructivist] fluff’. Different coordinate representations of the same physical configuration, different proofs of the same proposition, different constructions of the same mathematical object, different senses of the same reference, different intentional presentations of the same extensional concept, all these differences that seem to make no difference are considered as nothing but representational artefacts that we should carefully distinguish from the intrinsic properties of the objects at stake.

On the other side, there is a tradition—that can be traced back at least to Leibniz and Galois—that accepts at face value the existence of indiscernibles in physics and mathematics. According to this tradition, the problem posited by the existence of indiscernible entities cannot be solved by trying to eliminate indiscernibilities—by introducing different grades of discernibility a la Quine, by assuming the existence of hidden discerning properties, by removing gauge-dependent quantities or by truncating the homotopic structure—but rather by introducing new mathematical formalisms capable of encoding the very structure carried by indiscernibles. The main motivation to do so is that in certain situations the truncation of this ‘surplus structure’ leads to different kinds of pathological constructions, like the so-called ‘bad quotients’ associated with group actions that are not free, or the problems encountered when trying to define *moduli spaces* for objects with non-trivial automorphisms in algebraic geometry. On this side of the divide, progress does not move in the direction of removing the differences that make no difference, but rather in the direction of ‘resolving’ the mathematical structure that they present.

It is also worth noting that the question of identity plays a key role in the different strategies intended to hold space for indiscernibles that we have briefly described. According to the authors that advocate an ontology and a logic of non-individuals, indiscernibles can be introduced by suspending the universal application of the self-identity predicate x=a. We could say that in this approach, the notions of *no-identity*, *non-individuality* and *indiscernibility* go together. By contrast, the mathematical approaches based on homotopy type theory seem to hold space for indiscernibles (a) by expanding or stretching the mathematical notion of equality beyond strict equality and (b) by understanding equalities as types of proofs. In this framework, an entity a:X might have a non-trivial identity in the sense that there might be different inequivalent proofs of the proposition $a{=}_{X}a$ (this is typically the case when a has non-trivial symmetries or automorphisms). In this way, the attempts to hold space for indiscernible entities seems to lead either to *entities without identity* or to *entities with a non-trivial identity*. The common point is that in both cases it seems necessary to go beyond set-theoretic foundations in order to cope with indiscernible entities. That being said, the scope of this comparison is limited by the fact that the corresponding notions of indiscernibility are not necessarily the same. Whereas the notion of non-individual was forged to deal with quantum indiscernibility, the canonical example of indiscernibility formalized by homotopy type theory is the indiscernibility between two path-connected points in a space. It is also worth noting that the project of going beyond set-theoretic foundations in order to hold space for indiscernible objects does not depend on any ontological thesis about the ultimate nature of the corresponding indiscernibilities. Even if indiscernible objects arise as a result of a process of abstraction (as Leibniz claimed [36, p. 32]) or as a consequence of the limitations of the corresponding language (as it is the case in Galois theory), or if they are objects that *emerge* in certain regimes or under certain approximations, once they are there they present a rich mathematical structure that has to be properly understood.

Independently of the reader’s position with respect to the understanding of indiscernibilities in physics, mathematics and philosophy, there is no doubt that the discussions around these topics provide important and currently active vectors of innovation in these different disciplines.

## Data Availability

This article has no additional data.

## Footnotes

1 In what follows we shall use the terms *indistinguishability* and *indiscernbility* as synonyms. In certain domains (like quantum theory), the former is generally used, while in others (e.g. the philosophical discussions about Leibniz’s principle of the identity of indiscernibles) the latter is preferred.

2 ‘Since any definition is an identity, identity itself cannot be defined’ [5, p. 80].

3 Very briefly, the existence of elements in a structure with the same structural properties (i.e. of elements that entertain the same relations with other elements) seems to be an obstacle to the thesis that the different elements in a structure can be completely individualized by specifying their structural properties.

4 For instance, Russell writes: ‘The particular cannot be defined or recognized or known; it is something serving the merely grammatical purpose of providing the subject in a subject-predicate sentence such as ‘this is red’. And to allow grammar to dictate our metaphysic is now generally recognized to be dangerous. It is difficult to see how something so unknowable as such a particular would have to be can be required for the interpretation of empirical knowledge. The notion of a substance as a peg on which to hang predicates is repugnant’ [29, Part IV, Ch. 8].

5 When we say ‘two of them’, we are using the fact that in many circumstances it is physically meaningful to say that quantum field modes have well-defined occupation numbers. Therefore, assertions such as ‘there are two quanta in a given state’ are meaningful. However, it is worth stressing that this does not mean that we are necessarily endorsing an ontology of particles (for a clear conceptual analysis of the distinction between the notions of *particle* and *quanta* see [79, Chap. 2, pp. 16–36]).

6 This assumption of continuity for macroscopic objects has been described by Schrödinger in the following terms: ‘If a familiar article, say an earthenware jug, disappears from your room, you are quite sure that somebody must have taken it away. If after a time it reappears, you may doubt whether it really is the same one—breakable objects in such circumstances are often not. You may not be able to decide the issue, but you will have no doubt that the doubtful sameness has an indisputable meaning—that there is an unambiguous answer to your query. So firm is our belief in the continuity of the unobserved parts of the string!’ [85].

7 It is worth quoting Schrödinger at length: ‘Democritus and all who followed on his path up to the end of the nineteenth century, though they had never traced the effect of an individual atom [⋯], were yet convinced that the atoms *are* individuals, identifiable, small bodies just like the coarse palpable objects in our environment. It seems almost ludicrous that precisely in the same years or decades which let us succeed in tracing single, individual atoms and particles, and that in various ways, we have yet been compelled to dismiss the idea that such a particle is an individual entity which in principle retains its ‘sameness’ for ever. Quite the contrary, we are now obliged to assert that the ultimate constituents of matter have no ‘sameness’ at all. When you observe a particle of a certain type, say an electron, now and here, this is to be regarded in principle as an *isolated event*. Even if you do observe a similar particle a very short time later at a spot very near to the first, and even if you have every reason to assume a *causal connection* between the first and the second observation, there is no true, unambiguous meaning in the assertion that it is *the same* particle you have observed in the two cases. [⋯] And I beg to emphasize this and I beg you to believe it: It is not a question of our being able to ascertain the identity in some instances and not being able to do so in others. It is beyond doubt that the question of ‘sameness’, of identity, really and truly has no meaning.’ [87, pp. 121–122].

8 In Manin’s words: ‘I would like to point out that this is rather an extrapolation of commonplace physics, where we can distinguish things, count them, put them in some order, etc. New quantum physics has shown us models of entities with quite different behaviour. Even ‘sets’ of photons in a looking-glass box, or of electrons in a nickel piece are much less Cantorian than the ‘set’ of grains of sand’ [88, p. 36].

9 This point was stressed by Bigaj and Ladyman in the following terms: ‘Of course, PII is trivial if the way in which the things differ is in being distinct. Hence, the true metaphysical significance of the principle is arrived at if it is true even when restricted to qualitative or non-identity-involving properties [⋯]’ [52].

10 For other discussions regarding the applicability of the notion of ‘primitive thisness’ to quantum systems see [100–102] and [79, pp. 16–35], as well as Kastner [99].

11 By pushing further this similarity between exchange symmetries and gauge symmetries, Margeneau argues that Pauli’s exclusion principle, ‘by merely stipulating antisymmetry, automatically introduces correlations between the states of the two particles. Although the correlations are of non-dynamical origin, arising as they do a formal principle of symmetry, they have the same physical effects as if were due to forces [⋯] There is an interesting and far-reaching parallelism between the general principle of relativity and the exclusion principle. The former creates physically perceptible forces out of the metric of space; by endowing its equations with the formal property of invariance it is able to account for the phenomenon of gravitation, no reference being made to the ordinary concept of force. The exclusion principle imposes another formal property, antisymmetry, upon the state functions of quantum physics and thereby yields correlations which are tantamount to forces. The physicist in fact calls them *exchange forces* without any apparent embarrassment’ [64, pp. 195–196].

12 This position could be traced back to Kretschmann’s objection against the supposed physical scope of the principle of general covariance in general relativity [123].

13 Briefly, the gauge principle encodes the heuristic fact that the a global symmetry of a Lagrangian can be made local by adding a gauge field, that is, a potential of a physical interaction.

14 According to Rovelli, ‘[g]auge invariance is not just mathematical redundancy; it is an indication of the relational character of fundamental observables in physics. These do not refer to properties of a single entity. They refer to relational properties between entities: relative velocity, relative localization, relative orientation in internal space and so on. Gauge interactions describe the world because Nature is described by relative quantities that refer to more than one object. [⋯] Gauge is ubiquitous. It is not unphysical redundancy of our mathematics. It reveals the relational structure of our world’ [125].

15 In Greaves and Wallace’s terms, ‘a transformation can be a symmetry of a subsystem of the world without being a symmetry of the whole world. As such, the transformation gives rise to a different state of affairs, but has no detectable consequences for measurements confined to the subsystem’ [118, p. 60].

16 This point has been clearly stressed by Maudlin as follows ‘[⋯] the notion of [⋯] an inertial frame of reference, is derivative rather than fundamental. Each of these concepts can only be defined by reference to some objective geometrical structure of space–time itself, in order to make sense of the qualifier ‘inertial’. So we ought to begin with the intrinsic geometry, not with coordinate systems or reference frames’ [141, p. 67].

17 According to Henneaux and Teitelboim, ‘It is a remarkable occurrence that the road to progress has invariably been toward enlarging the number of variables and introducing a more powerful symmetry rather than conversely aiming at reducing the number of variables and eliminating the symmetry’ [111, p. xxiii].

18 See Muller [142] for an analysis of the notion of identity in Aristotle’s philosophy and the history of the equal sign ‘=’.

19 It is worth noting that, in the framework of Galois theory, the K-indiscernibilities at stake are purely epistemic rather than intrinsic, since they can be broken by passing to a larger field $K^{\prime }⊃K$ endowed with a higher ‘resolving power’. What cannot be discerned in a given arithmetic language (the field K) can be discerned in a field extension of that language. The so-called *Galois correspondence* encodes the correspondence between different arithmetic languages (field extensions $K^{\prime }⊃K$) and the corresponding *Galois groups* of indiscernibilities.
